# GDF15 Regulates Malat-1 Circular RNA and Inactivates NFκB Signaling Leading to Immune Tolerogenic DCs for Preventing Alloimmune Rejection in Heart Transplantation

**DOI:** 10.3389/fimmu.2018.02407

**Published:** 2018-10-30

**Authors:** Yixin Zhang, Guangfeng Zhang, Yanling Liu, Renqi Chen, Duo Zhao, Vivian McAlister, Tina Mele, Kexiang Liu, Xiufen Zheng

**Affiliations:** ^1^Departments of Cardiovascular Surgery, Jilin University, Changchun, China; ^2^Department of Pathology, Western University, London, ON, Canada; ^3^Department of Rheumatology, Guangdong Academy of Medical Sciences, Guangdong General Hospital, Guangzhou, China; ^4^Division of General Surgery, Department of Surgery, Western University, London, ON, Canada; ^5^Department of Oncology, Western University, London, ON, Canada; ^6^Lawson Health Research Institute, London, ON, Canada; ^7^London Health Sciences Centre, London, ON, Canada

**Keywords:** GDF15, circular RNA, circ_Malat-1, tolerogenic DCs, heart transplantation, immune suppression, tolerance

## Abstract

Recombinant human growth differentiation factor 15 (rhGDF15) affects dendritic cell (DC) maturation. However, whether GDF15 is expressed in DCs and its roles and signaling in DCs remain largely unknown. It is unclear whether GDF15-DCs can induce immune tolerance in heart transplantation (HT). This study aims to understand the impact of endogenous GDF15 on DC's development, function, underlying molecular mechanism including circular RNA (circRNA). This study will also explore GDF15-DC-mediated immune modulation in HT. Bone marrow (BM) derived DCs were cultured and treated to up- or down regulate GDF15 expression. Phenotype and function of DCs were detected. Expression of genes and circRNAs was determined by qRT-PCR. The signaling pathways activated by GDF15 were examined. The impact of GDF15 treated DCs on preventing allograft immune rejection was assessed in a MHC full mismatch mouse HT model. Our results showed that GDF15 was expressed in DCs. Knockout of GDF15 promoted DC maturation, enhanced immune responsive functions, up-regulated malat-1 circular RNA (circ_Malat 1), and activated the nuclear factor kappa B (NFκB) pathway. Overexpression of GDF15 in DCs increased immunosuppressive/inhibitory molecules, enhanced DCs to induce T cell exhaustion, and promoted Treg generation through IDO signaling. GDF15 utilized transforming growth factor (TGF) β receptors I and II, not GFAL. Administration of GDF15 treated DCs prevented allograft rejection and induced immune tolerance in transplantation. In conclusion, GDF15 induces tolerogenic DCs (Tol-DCs) through inhibition of circ_Malat-1 and the NFκB signaling pathway and up-regulation of IDO. GDF15-DCs can prevent alloimmune rejection in HT.

## Introduction

Heart transplantation (HT) is the gold standard for the treatment of patients with end stage heart failure or severe coronary artery disease. Despite improvements in short-term outcomes, long-term patient and graft survival remain suboptimal due to the toxic side effects associated with long-term use of immunosuppressive drugs. An ideal treatment able to induce allograft-specific tolerance in transplant recipients, which enables long-term graft survival while avoiding the need for immunosuppression and its associated adverse effects, is urgently needed. More detailed elucidation of new immune suppressive molecules and signaling pathways will help develop innovative new immune rejection treatment strategies for HT.

Dendritic cells (DCs) are highly specialized and functionally diverse antigen-presenting cells (APCs) and are responsible for the control of the innate and adaptive immune responses. Originating from bone marrow (BM) progenitor cells, DCs can differentiate into immune responsive or immune suppressive/tolerogenic, depending on stimulus. In organ transplantation, DCs play a crucial role in determining the fate of a transplanted organs: rejection or acceptance by a host, depending on their immune suppressive or immune responsive function (1). Specifically, immune tolerogenic DCs (Tol-DCs) specifically are positively related to allograft survival and in nonhuman primates, have been shown to prolong allograft survival in a variety of organ transplantation models (2). In contrast, immunogenic DCs initiate graft rejection. Tol-DCs could be induced by many strategies including treatment with maturation-inhibiting agents, (3) blockade of costimulatory molecules with antibodies (4) and siRNA (5) as well as pre-treatment with immunosuppressant (6). We previously demonstrated that *in vitro* generated Tol-DCs could prevent allograft rejection in animal HT models (6–11), implying that Tol-DCs might be an ideal treatment for transplantation. Nevertheless, immune tolerance induction still needs to be further explored and improved.

GDF15, also named as macrophage inhibitor cytokine (MIC-1), is a divergent member of the TGF-β superfamily, and is associated with many diseases including cardiovascular disease and cancer (10–12). It displays immune suppressive function (12–14). Zhou et al. reported that treatment with recombinant GDF15 (rhGDF15) suppresses expression of surface molecules CD83, CD86, and HLA-DR in DC, thereby preventing the recruitment of T cells leading to acceleration of tumor growth in a cancer model (15). It suggests that GDF15 might be a good candidate for preventing immune rejection in HT. However, it is unknown yet whether DCs express GDF15 and what roles it plays in DC development and by what molecular mechanism(s) they function. Whether GDF15-modulated DCs can prevent transplant hearts (allografts) from immune rejection in HT remains to be addressed.

Moreover, recently emerging evidence is showing that a new class of endogenously expressed non-coding RNAs named as circular RNAs (circRNAs) regulate gene expression and function (16, 17). circRNAs are produced from back splicing and form a covalently closed loop without free terminals (18). CircRNAs play critical roles in physiological and pathological processes due to their special structure, abundance, conservation across species, and functions. However, there are no reports about circRNAs in DC development and any associatations between GDF15 and circRNA are unknown.

In this study, we aimed to investigate the role of endogenous GDF15 in DC development and DC-mediated immune tolerance induction, and signaling pathways activated by GDF15. We also aimed to reveal the involvement of circRNA in DC development and association with GDF15 in DCs and to determine the impact of GDF15-regulated DCs in preventing allograft rejection and immune tolerance induction in HT.

## Materials and methods

### Animals

C57B/6 wild type mice and BALB/c mice were purchased from Charles River Laboratories (Charles River Canada, Saint-Constant, Canada). Whole genome GDF15 knock-out (KO) mice, generated on a C57BL/6 using standard gene-targeting techniques, were kindly provided by Professor Se-Jin Lee at John Hopkins University (Baltimore, MD). GDF15 Transgenic (TG) mice ubiquitously expressing high levels of human GDF15 (hNAG1) under the control of the chicken β-actin promoter (CAG) were kindly provided by Dr. Seung J. Baek at the University of Tennessee (Knoxville, TN, USA) (19). Male C57BL/6 (H-2b) and BALB/c (H-2d) mice at the age of 9–10 weeks were used as donors and recipients, respectively. Animals were housed at the Conventional Animal Care Facility, University of Western Ontario, and were cared for in accordance with the guidelines established by the Canadian Council on Animal Care. All animal experiments in this study were approved by the Committee of Animal Use of the University of Western Ontario.

### DC culture

Bone marrow (BM) derived DCs were cultured from BM progenitor cells as previously described (9). Briefly, BM cells were flushed from the femurs and tibias of C57BL/6, GDF15 KO, or GDF15 TG mice, washed and cultured in 6-well plates in the presence of 10 ng/ml of recombinant GM-CSF and recombinant mouse IL-4 (Peprotech, Rocky Hill, NJ) at 37°C in 5% humidified CO_2_. Non-adherent cells were removed (Day 2) and fresh medium was added. Half of the medium was replaced every 2 days.

### DC transfection and infection

Day 5 cultured BM derived DCs (1 million/well) were plated in a 12 well plate and transfected with 1 μg GDF15 siRNA using lipofectamine 2000 (Life technologies, Burlington, Canada) at the ratio of 1:2 for 4 h. Twenty-four hours after transfection, LPS (50 ng/ml) was added to the medium for 3 h and then the cells were re-collected for further experiments.

Infection of DCs with GDF15 expression adenovirus was conducted on day 2. Day 2 cultured BM-derived DCs (2 million/well) were plated in a six well plate and infected with human GDF15 expression adenovirus (GDF15-Ad) or Null-Ad SignaGen Laboratories, Gaithersburg, MD 20884-0661) with multiplicity of infection (MOI) of 100 in 1 ml medium for 6 h. Six hours after infection, fresh additional 3 ml of DC medium was added to the DCs and allowed to continue culture. Half of the culture medium was replaced every other day, until the time for the next experiments.

### Quantitative reverse-transcriptase polymerase chain reaction (qRT-PCR)

Total RNA was extracted from cells and tissues using Trizol (Invitrogen). cDNA was synthesized from RNA using oligo-(dT) primer and reverse transcriptase (Invitrogen). Primers used for the amplification of murine GDF15, IDO 1, IDO2, IL-2, IL-1β, RelA, Rel B, PD-1, TIM-3, PD-L1, BTLA, GFRAL, and GAPDH genes are listed in Supplemental Table 1. qPCR was conducted in the CFX connect^TM^ Real Time System (BioRad, Mississauga, Ontario) or Stratagene Mx3000P QPCR System (Agilent Technologies, Lexington, MA) using SYBR green PCR Master Mix (Froggabio Inc., ON, Canada) and 100 nM of forward and reverse primers. The PCR condition was 95°C for 2 min, 95°C for 10 s, 58°C for 10 s, and 72°C for 20 s (40 cycles).

### Circular RNA malat-1 expression and sequencing

cDNA was synthesized from total RNA using hexamer random primers and reverse transcriptase according to instruction of the manufacturer (Invitrogen). Divergent primers spanning the junction of circ-Malat-1 (Forward: gcctttggcctaatcacaga; and Reverse: ttgtggggagaccttgaaac) were designed and used for qPCR. qPCR was conducted in the CFX connect^TM^ Real Time System (BioRad) using SYBR green PCR Master Mix (Froggabio Inc.) and 100 nM of forward and reverse primers. The PCR condition was 95°C for 2 min, 95°C for 5 s, 60°C for 10 s, and 72°C for 10 s (40 cycles).

Regular PCR using Taq DNA polymerase was conducted to amplify the circ-Malat-1 fragment. The amplified fragment was subjected to DNA sequencing at The Robarts Research Institute in London Ontario, Canada.

### Flow cytometry

Flow cytometry was performed to characterize the phenotype of DCs and T cells using a Calibur flow cytometer (Becton Dickinson, San Jose, CA) or Cyto Flex S (Beckman). Antibodies were purchased from eBioscience, San Diego, CA.

DC and T cell subsets were analyzed by means of two- or three-color staining with various combinations of mAbs. DCs were stained with FITC- or PE-CD86, FITC- or PE-CD11C, PE-CD80, PE or APC CD-83, FITC-MHCII, PE-PD-L1, and PE-Cy5-CD40 monoclonal antibodies. For T cells, PE-Cy5-CD4, PE-CD25 and FITC-FoxP3, Percp-efluor710-PD-1, APC-BTLA, and PE-TIM 3 conjugated anti-mouse monoclonal antibodies were used for staining. Foxp3 expression was assessed by intracellular staining, using a cell permeabilization kit (eBioscience). Appropriate isotype controls were included.

### Western blotting

DCs were collected and washed with PBS. Total proteins were extracted with RIPA buffer containing protease inhibitor MSCF followed by three cycles of 5 s sonication. For heart tissue, 10 mg of heart tissues were homogenized with PRIPA buffer containing protease inhibitor MSCF using manual homogenizer on ice prior to sonication. Cell lysate and tissue lysate was centrifuged for 20 min at 15,000 rpm and supernatant was collected. The concentration of protein was measured using the Bradford method with Bradford 1 × Dye reagent (BioRad, Mississauga, Ontario, Canada). Twenty micrograms total protein was loaded on 12% polyacrimide gel and run for 60–90 min at 100 V. Separated proteins were transferred to PCM membrane. Transferred membranes were blocked with 5% fat-free milk powder in TBST for 30 min at room temperature and then blotted with the primary antibodies against mouse or human GDF15,TGFβ RI, and TGFβ RII (1:1,000 dilution, Sigma), phosphorylated Rel A p65, (1:1,000 dilution, Cell Signaling Technology, Danvers, MA), total Rel A p65 (1:1,000 dilution, Cell Signaling Technology), and β-actin (1:4,000 dilution, Santa Cruz Biotechnologies, San Diego, CA) at 4°C for overnight. The blotted membranes were washed with TBST containing 0.25% Tween-20 for 10 min at room temperature and repeatedly washed for three times. Washed membranes were blotted with appropriated second antibodies (Santa Cruz Biotechnologies) for 30 min at room temperature. Proteins were developed with ECL kits (Bio-Rad, Hercules, CA 94547) and visualized by FluorChem M system (ProteinSimple, San Jose, CA).

### Immunoprecipitation assays

DCs were collected and washed with cold PBS and lysed with RIPA buffer containing protease inhibitor MSCF for 5 min on ice. Cell lysate was centrifuged at 10,000 rpm for 5 min at 4°C. Supernatant was collected and protein concentration was measured using the Bradford method with Bradford 1X Dye reagent (Bio-Rad). Two hundred micrograms total proteins were used and 2 μg GDF15 Abs (K-13, Santa Cruz) were added to the protein and incubated at 4°C overnight. 18 μl of Protein A/G plus Agarose IP reagent beads (Santa Cruz) were added to each sample and incubated at 4°C for 3 h. The mixture was centrifuged at 2,500 × g for 5 min at 4°C. The pellets were washed with 1 ml PBS for three times. After the final cycle of washing, 20 μl of PBS and 10 μl 6X loading buffer was added to each sample. The suspended sample was boiled at 95°C for 5 min to denature protein and spanned down at 2,500 × g at 40°C for 1 min. Denatured samples were subjected to Western blotting.

### Mixed lymphocyte reaction (MLR)

A mixed lymphocyte reaction was conducted to measure T cell proliferation. T cell proliferation was measured using a carboxyfluorescein succinimidyl ester (CFSE) dilution assay *in vitro* (20).

For a CFSE dilution assay, T cells (2 × 105/well) from naïve BALB/c mice were labeled with CFSE and then co-cultured with allogeneic BM-derived DCs cultured from C57BL/6 mice at a ratio of DC:*T* = 1:5 cells in 200 μl of complete RPMI 1640 medium (Life Technologies). Cells were cultured at 37°C in a humidified atmosphere of 5% CO_2_ for 3 days. The dilution of CFSE was measured by flow cytometry with a Calibur flow cytometer (Becton Dickinson, San Jose, CA).

### Enzyme-linked immunosorbent assay (ELISA)

Levels of IFN-γ, TGFβ, and IL-10 in DC the culture medium were determined using mouse IFNγ, TGFβ, and IL-10 Quantikine ELISA kit (R&D Systems) according to the manufacturer's protocol. Optical density values were measured at 450 nm on an ELISA plate reader.

### Heterotopic cardiac transplantation

Recipient mice (BALB/c) were intravenously treated with 1 million of DCs infected with human GDF15 expressing adenovirus through the tail vein of mice seven days prior to HT. Recipient BALB/c mice were anesthetized with Ketamine/Xylene and subjected to intra-abdominal allogeneic cardiac transplantation using the hearts from fully MHC-mismatched C57BL/6 mice according to our well-established procedure (10). After transplantation, recipients daily received sub dose rapamycin (1 mg/kg) through intra-peritoneal (*i.p*.) injection from days 0 to 7. Pulsation of implanted heart grafts was monitored daily by direct abdominal palpation in a double-blind manner to determine graft rejection/survival.

### Statistical analysis

In this study, data were reported as the mean ± SEM. Quantitative real-time PCR data were analyzed using one-way ANOVA or student's *t*-test. Allograft survival among experimental groups was compared using the log-rank test. Differences with *P*-values < 0.05 were considered significant.

## Results

### GDF15 is expressed in DCs and up-regulated by exogenous rhGDF15

**Figure 1:**
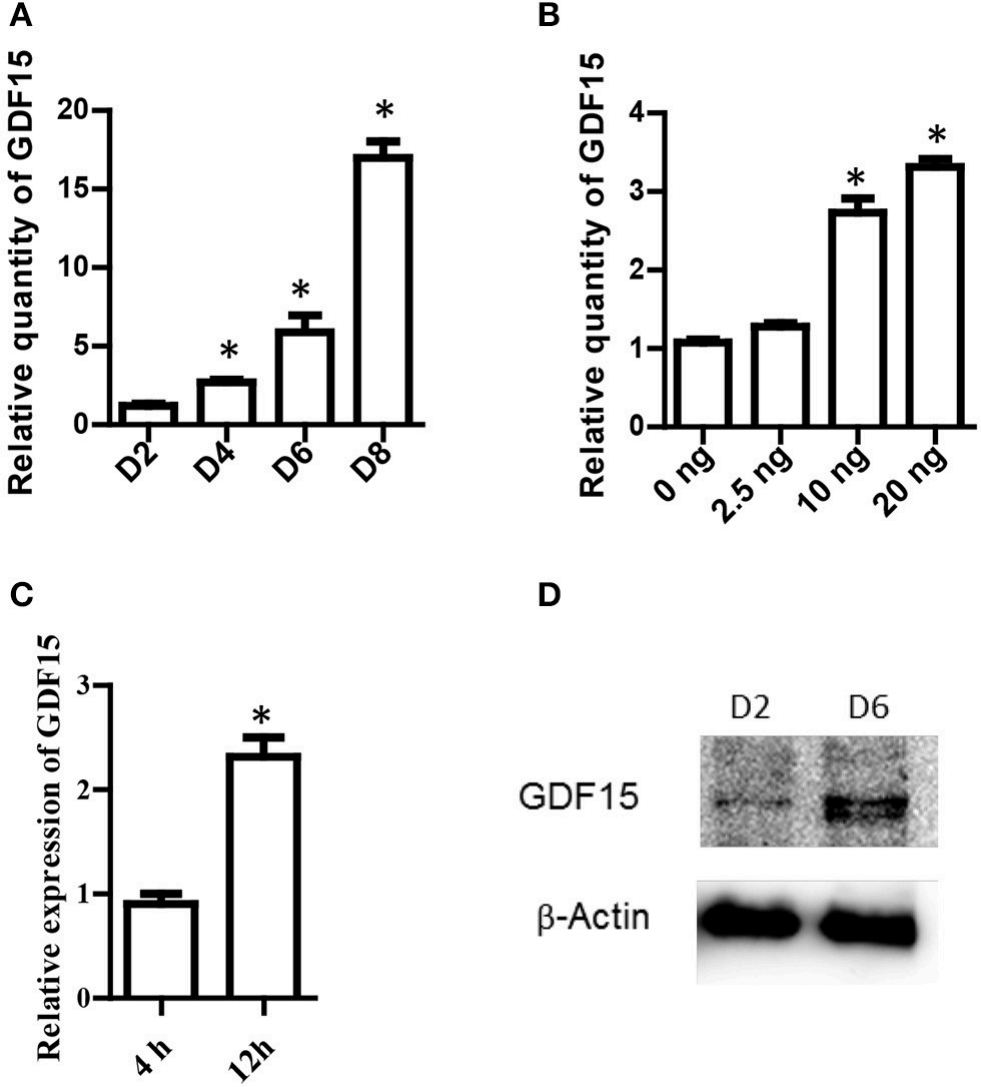
GDF15 expression in DCs. **(A)** A time course of GDF15 expression in DCs. DCs (10^6^ cell/well) were cultured from bone marrow progenitors in the presence of IL-4 and granulocyte/macrophage colony stimulating factor (GM-CSF). Cultured DCs were collected on day 2, 4, 6, and 8 for RNA extraction. The level of GDF15 mRNA was detected by qRT-PCR and GAPDH was used as loading control. Relative quantity of GDF15 mRNA was the expression of GDF15 in Day 2 DCs. *n* = 4, One way ANOVA was conducted for statistical analysis. **(B)** rhGDF15 increased GDF15 expression in DCs. BM-derived DCs were cultured and a variety of concentrations of rhGDF15 were added on day 4. Twenty-four hours after treatment, GDF15 expression in DCs was detected using qRT-PCR. GDF15 expression was normalized with control DCs without rhGDF15 treatment. *n* = 4, One way ANOVA was conducted for statistical analysis. **(C)** The up-regulation of GDF15 in DCs by rhGDF15 was time related. BM-derived DCs were treated with 2.5 ng/ml rhGDF15 on day 5. *n* = 3, the student *T*-test was conducted for statistical analysis. GDF15 expression in DCs was detected 4 h and 12 h after addition of rhGDF15 by qRT-PCR. The expression of GDF15 in DCs treated for 4 h was used as a normalizer. **(D)** GDF15 protein expressed in DCs. Total protein was extracted from day 2 and 6 *in vitro* cultured DCs. GDF15 expression at the protein levels was determined by western blotting using GDF15 Abs. β-Actin was used as a loading control. Images were representatives of *n* = 3, *P < 0.05.

It has been reported that exogenous rhGDF15 affects DC maturation and function (15). However, it remains unknown whether DCs themselves express GDF15 and whether exogenous rhGDF15 affects endogenous GDF15 expression in DCs. To address these questions, BM-derived DCs from wild type C57BL/6 mice were cultured *in vitro* with RPIM1640 in the presence of GM-CSF and IL4. RNA were extracted from DCs on different culture days. GDF15 expression in DCs was measured by qRT-PCR. As shown in Figure 1A, GDF15 was detectable on day 2 of the culture and its expression levels gradually increased over time.

Next, rhGDF15 was added to DC culture at various concentrations on different days of culture to determine the effect of exogenous rhGDF15 on GDF15 expression in DCs. Addition of exogenous rhGDF15 up-regulated the expression of GDF15 in DCs, and the increase of expression levels was both dose (Figure 1B) and treatment duration dependent (Figure 1C). GDF15 expression in DCs was also confirmed at the protein level by Western blotting (Figure 1D).

### Deficiency of GDF15 promotes DC maturation

**Figure 2:**
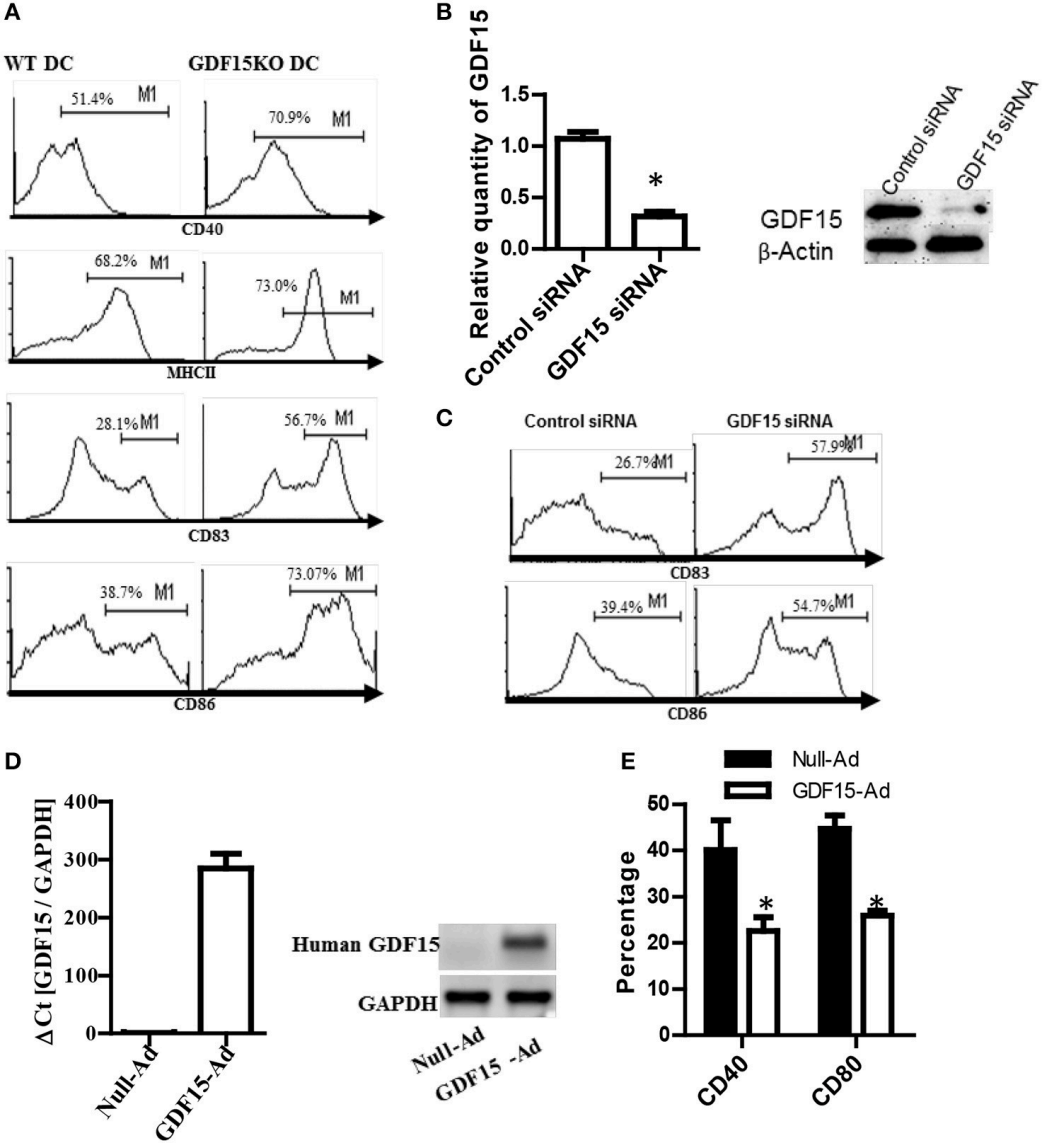
Knockout of GDF15 promoted DC maturation. **(A)** GDF15 KO DCs promoted DC maturation. BM-derived DCs were cultured from GDF15 KO and WT mice. On day 6, DCs were stimulated with 50 ng/ml LPS for 3 h. DC maturation was detected by measuring the expression of MHCII, CD40, CD83, and CD86 using flow cytometry. Images were representatives of *n* = 4. **(B)** GDF15 silenced GDF15 expression in DCs. Day 5 DCs cultured from WT mice were transfected with GDF15 siRNA for 24 h. GDF15 expression was determined by qRT-PCR (left) and Western blotting (right). *n* = 3, the student *T*-test was conducted for statistical analysis of qRT-PCR results. **(C)** GDF15 siRNA increased the expression of CD83 and CD86 in DCs. DCs were transfected with GDF15 siRNA. 24 h after transfection, CD83 and CD86 expression was detected by flow cytometry. Data were representatives of *n* = 3. **(D)** GDF15-Ad increased GDF15 expression in DCs. Day 4 DCs were infected with 100MOI of adenovirus expressing human GDF15 qRT-PCR (left, *n* = 3) and Western blotting (right). Data were representatives of *n* = 3. **(E)** GDF15-Ad decreased CD40 expression in DCs measured by flow cytometry. *n* = 3, the student *T*-test was conducted for statistical analysis. *P < 0.05.

To study the impact of endogenous GDF15 on DC development, DCs were cultured from GDF15 KO and WT mice, respectively. The expression of MHCII and co-stimulatory molecules (CD40, CD83, and CD86), which are commonly used as DC maturation markers, was measured by flow cytometry and compared between groups. As shown in Figure 2A, GDF15 KO-DCs expressed significantly higher levels of MHCII, CD40, CD83, and CD86 than WT DCs, indicating that GDF15 deficiency promotes DC maturation.

To confirm the above effect of endogenous GDF15 on DCs development, DCs from WT C57BL/6 mice were transfected with siRNA specifically targeting GDF15 on day 5 to knock down GDF15 expression in DCs. 48 h after siRNA transfection, the expression of GDF15, CD86, and CD83 was detected by qRT-PCR, Western blotting and by flow cytometry, respectively. The results show that GDF15 siRNA effectively knocked down the expression of GDF15 expression at the mRNA levels (Figure 2B, left) and protein levels (Figure 2B, right). The expression of CD86 and CD83 was up-regulated in GDF15-siRNA transfected DCs as compared with control GL2 siRNA transfected DCs (Figure 2C), which is consistent with the result from KO DCs.

Furthermore, BM-DCs were cultured from WT C57BL/6 mice and then treated with adenovirus expressing the human GDF15 gene (GDF15-Ad) on day 2 *in vitro* to over-express GDF15. As expected, DCs infected with GDF15-Ad significantly over-expressed human GDF15 (Figure 2D). Over-expression of GDF15 resulted in a reduction of CD40 and CD80 (Figure 2E). Addition of rhGDF15 or GDF15-Ad to KO DCs reduced CD40 and CD80 compared to untreated KO DCs (Supplemental Figure 1). We also observed that DCs cultured from GDF15 TG mice presented the similar phenotype of DCS treated with rhGDF15 (data not shown). These data suggest that GDF15 expression level negatively correlates DC development and maturation.

### Lack of GDF15 enhances the capacity of DCs to activate T cells

**Figure 3:**
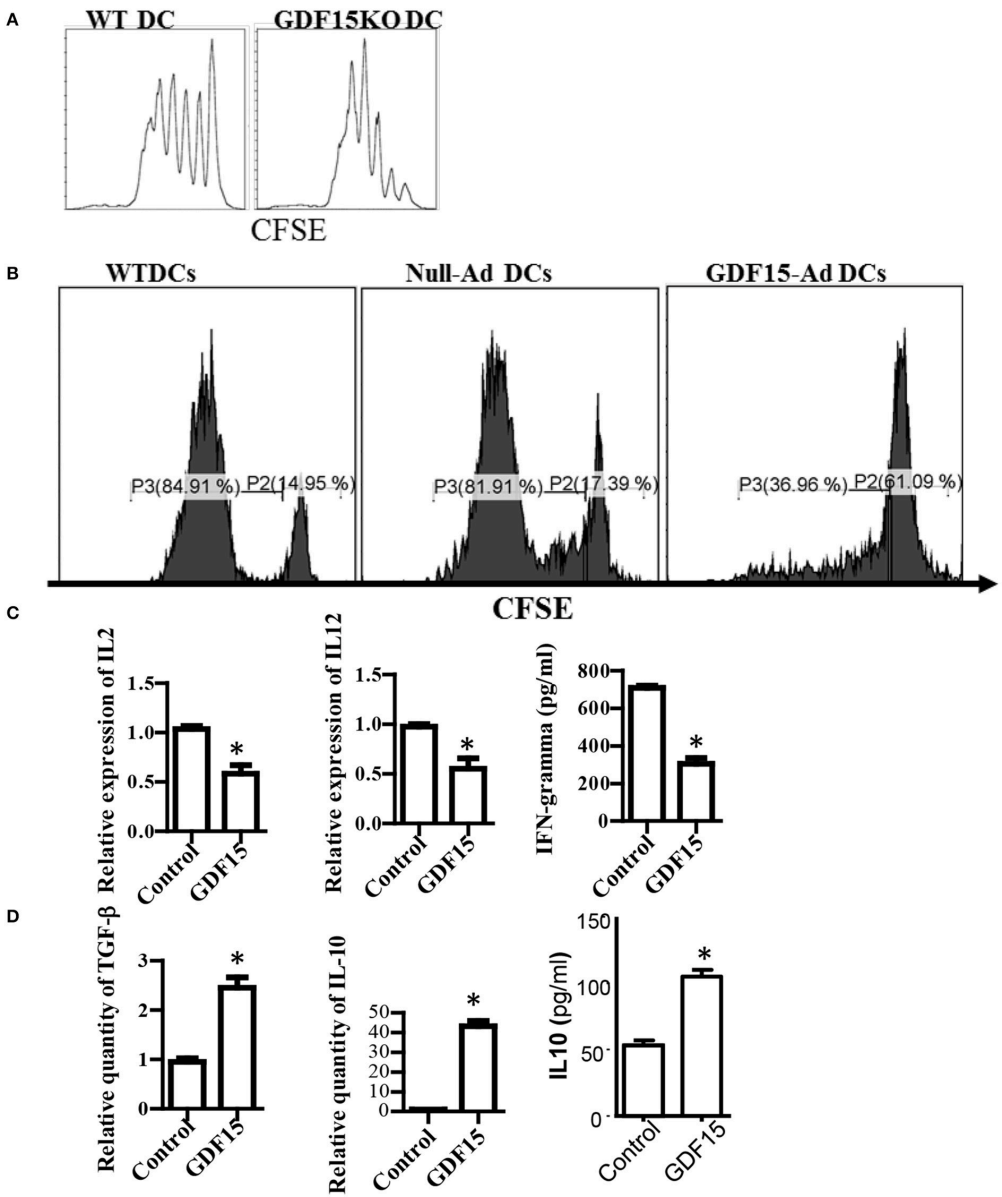
GDF15 impaired DC's capacity to activate T cells. **(A)** GDF15 KO DCs increased T cell proliferation. BM-derived DCs were cultured from WT or GDF15 KO mice. On day 6, an MLR was performed in which different cultured DCs were used as stimulators and allogeneic (BALB/c) CFSE-labeled T cells were used as responders. Proliferation was measured by CFSE dilution. Data were representatives of *n* = 3. **(B)** GDF15-Ad DCs inhibited T cell proliferation. BM-derived DCs were infected with GDF15-Ad on day 2 and an MLR was conducted to measure T cell proliferation**. **Data were representatives of *n* = 4. **(C)** GDF15 decreased the expression of proinflammatory genes. RNA was extracted from DCs treated with GDF15–Ad or Null-Ad. Expression of IL2 and IL-12 was detected by qRT-PCR and IFNγ in the culture medium was detected by ELISA. *n* = 3, the student *T*-test was conducted for statistical analysis. **(D)** GDF15 increased immunosuppressive cytokines IL10 and TGF-β. The expression of TGF-β and IL10 in the cells was detected by qRT-PCR and expression of IL10 in culture medium was detected by ELISA. *n* = 3, the student *T*-test was conducted for statistical analysis. *p < 0.05.

DCs are professional antigen presenting cells that are the unique cells able to activate naïve T cell response. To determine the effect of GDF15 on the capacity of DCs to activate naïve allogeneic T cells, an MLR was performed to measure T cell proliferation using a CFSE-dilution assay. As shown in Figure 3A, there was quicker dilution of CSFE labeled on T cells co-cultured with GDF15 KO DCs than those co-cultured with WT DCs, indicating that GDF15 deficiency enhances the capacity of DCs to activate naive allogeneic T cells. In contrast, the T cells co-cultured with GDF15 expressing adenovirus treated DCs proliferated less than those co-cultured with control DCs (Figure 3B). This data indicates that GDF15 attenuated DC function to activate naïve T cells, which is consistent with the result from Zhou et al. (15).

In addition, the expression of cytokines which are required for DCs to activate and polarize T cells was measured. Over-expression of GDF15 by infecti DCs with GDF15 expressing adenovirus decreased the expression of IL-2, IFN-g, and IL12p40 (Figure 3C), and increased the expression of TGF-β and IL10 (Figure 3D). Addition of rhGDF15 had similar results to GDF15 adenovirus (data not shown). The results indicated that GDF15 inhibited Th1-producing DCs that are characterized by high secretion of IL-12 and IFN-γ and low production of IL-10 (21).

### GDF15 promotes treg generation depending on IDO and increases immune checkpoint molecules

**Figure 4:**
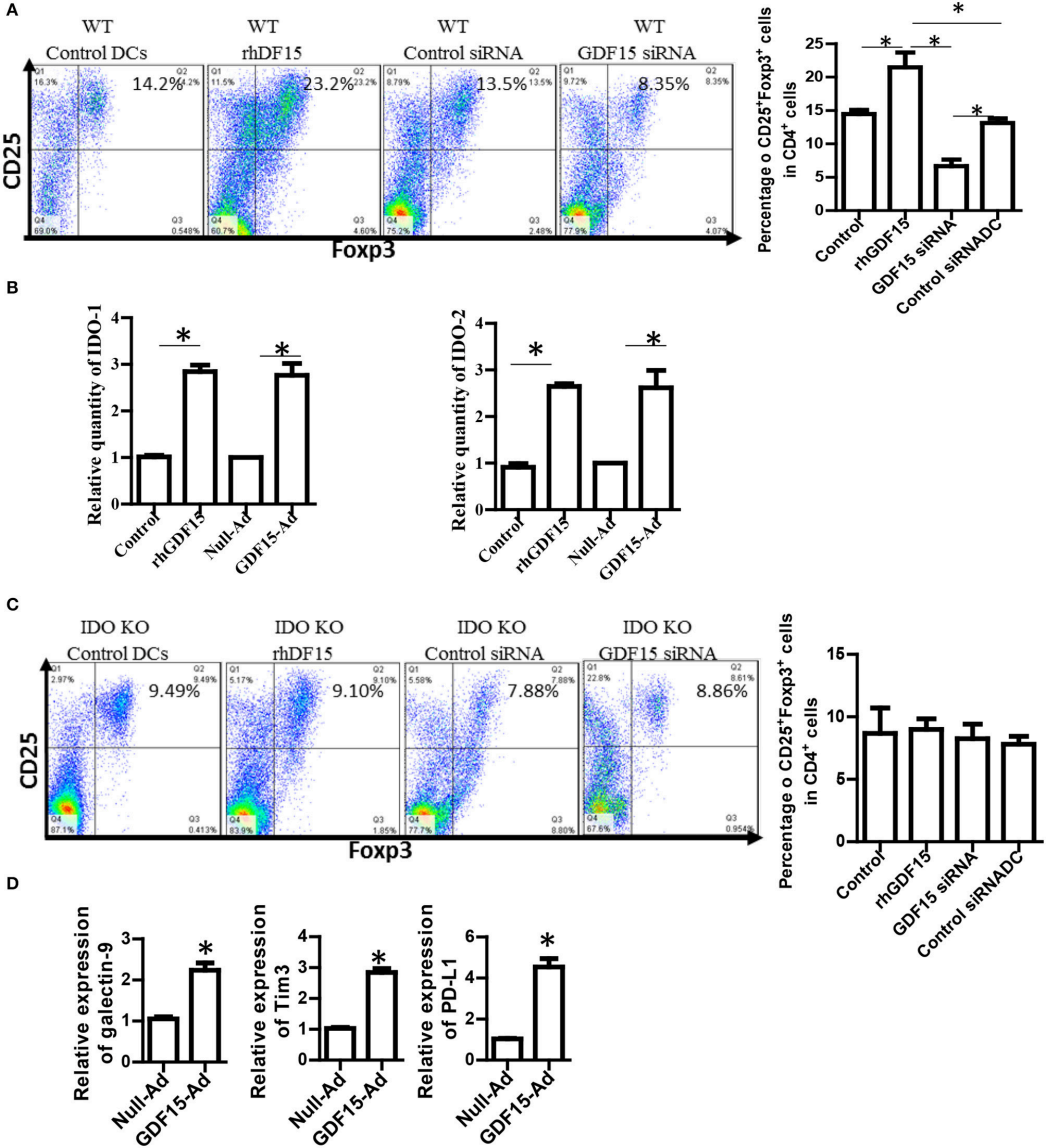
GDF15 enhanced DCs ability to generate Tregs. **(A)** The effect of GDF15 on Tregs. DCs were cultured from WT mice and treated with rhGDF15 or GDF15 siRNA, or control GL2 siRNA. rhGDF15 treated DCs and GDF15 silenced DCs or untreated DCs or GL2 siRNA transfected DCs were co-cultured with allogeneic naïve T cells from BABL/c mice at the ratio of 1:10 for 5 days. CD4^+^CD25^+^FoxP3^+^ cells were detected by flow cytometry. Representative images (upper) and summarized data (lower) from *n* = 5 experiments. The student *t*-test was conducted to compare data between rhGDF15 treated DCs and untreated DCs and between GDF15 siRNA and GL2 siRNA. *P < 0.05. **(B)** GDF15 increased the expression of IDO 1 and IDO2. DCs were cultured and treated with rhGDF15, GDF15 expression adenovirus (GDF15-Ad), or control null adenovirus (Null-Ad). Expression of IDO 1 and IDO2 in DCs was detected s by qRT-PCR, *n* = 3, the student *t*-test was conducted to compare data between rhGDF15 treated DCs and untreated DCs and between GDF15-Ad and Null-Ad. *P < 0.05. **(C)** Deficiency of IDO in DCs eliminated the effect of GDF15 on Treg generation. DCs were cultured from IDO KO mice and treated with rhGDF15 or GDF15 siRNA, then subjected to co-cultured with allogeneic T cells for 5 days. CD4^+^CD25^+^FoxP3^+^ cells were detected by flow cytometry. *P < 0.05. **(D)** GDF15 up-regulated immune inhibitory ligands. DCs were cultured and treated with GDF15-Ad or Null- Adenovirus. Expression of GDF15, Galectin 9, PD-L1 and TIM-3 was detected by qRT-PCR. Control adenovirus infected DCs were used as a normalizer. *n* = 3, the student *t*-test was conducted to compare data between Null-Ad and GDF15-Ad.*P < 0.05.

Generation of regulatory T cells (Treg) is one of the critical characteristics of Tol-DCs. To determine whether GDF15 induces Tol-DCs, the ability of GDF15 treated DCs to generate Tregs was studied. DCs with different levels of GDF15 were co-cultured with allogeneic naïve T cells from a BABL/c mouse at a ratio of DC: *T* = 1:10 *in vitro* for 5 days. CD4^+^CD25^+^FoxP3^+^ Tregs were measured by triple staining with CD4, CD25, and FoxP3 Abs followed by flow cytometry. 14% of CD4^+^CD25^+^FoxP3^+^ Tregs were detected in the T cells cultured with untreated WT DCs, 24% in rhGDF15 treated DCs, 13% in control siRNA transfected DCs, and 9% in GDF15 silenced DCs (Figure 4A), indicating that GDF15 promotes Tol-DCs to generate Tregs. We also found that DCs from GDF15 KO mice significantly reduced Treg generation while DCs from GDF15 TG mice significantly increased Treg, as compared to wild type DCs (Supplemental Figure 2).

It has been reported that IDO, an immunosuppressive molecule expressed in immunosuppressive DCs, plays a vital role in immune tolerance induction (22, 23) and is critical for DCs to generate Tregs (24). We proposed that GDF15 might enhance DCs to induce Treg through IDO. Therefore, IDO expression was first detected by qRT-PCR. As shown in Figure 4B, both rhGDF15 and GDF15-Ad treatment up-regulated the expression of IDO 1 (Figure 4B, left) and IDO 2 (Figure 4B, right) in DCs. Moreover, the expression levels of IDO in DCs positively correlated with the concentration of rhGDF15 (data now shown).

To verify whether IDO is involved in Treg induction by GDF15-DCs, BM-derived DCs was cultured from IDO KO mice and then treated with rhGDF15 or GDF15 siRNA 24 h after treatment. DCs were then subjected to a co-culture with allogeneic T cells for 5 days to produce Tregs. As shown in Figure 4C, the percentage of Tregs did not significantly change among the control, rhGDF15 treated DCs and GDF15 siRNA silenced DCs when IDO was knocked out as compared their controls, indicating GDF15 enhances Treg generation through up-regulation of IDO.

PD1 and PD-L1 signaling and TIM 3-galctin 9 signaling have been recently recognized as one of the important immune suppression mechanisms of DC-mediated immunosuppression (25). Overexpression of PD-L1 by DCs inhibited CD4+T cell activation (26). Accordingly, the expression of immune check point genes PD-1L, TIM3, and galectin 9 was detected. The results showed that over-expression of GDF15 increased PD-L1, TIM3, and galectin 9 expression (Figure 4D), suggesting GDF15 induced exhausted DCs.

### GDF15 reduces circular malat-1 (circ_malat-1) expression and inhibited the NFk b pathway

**Figure 5:**
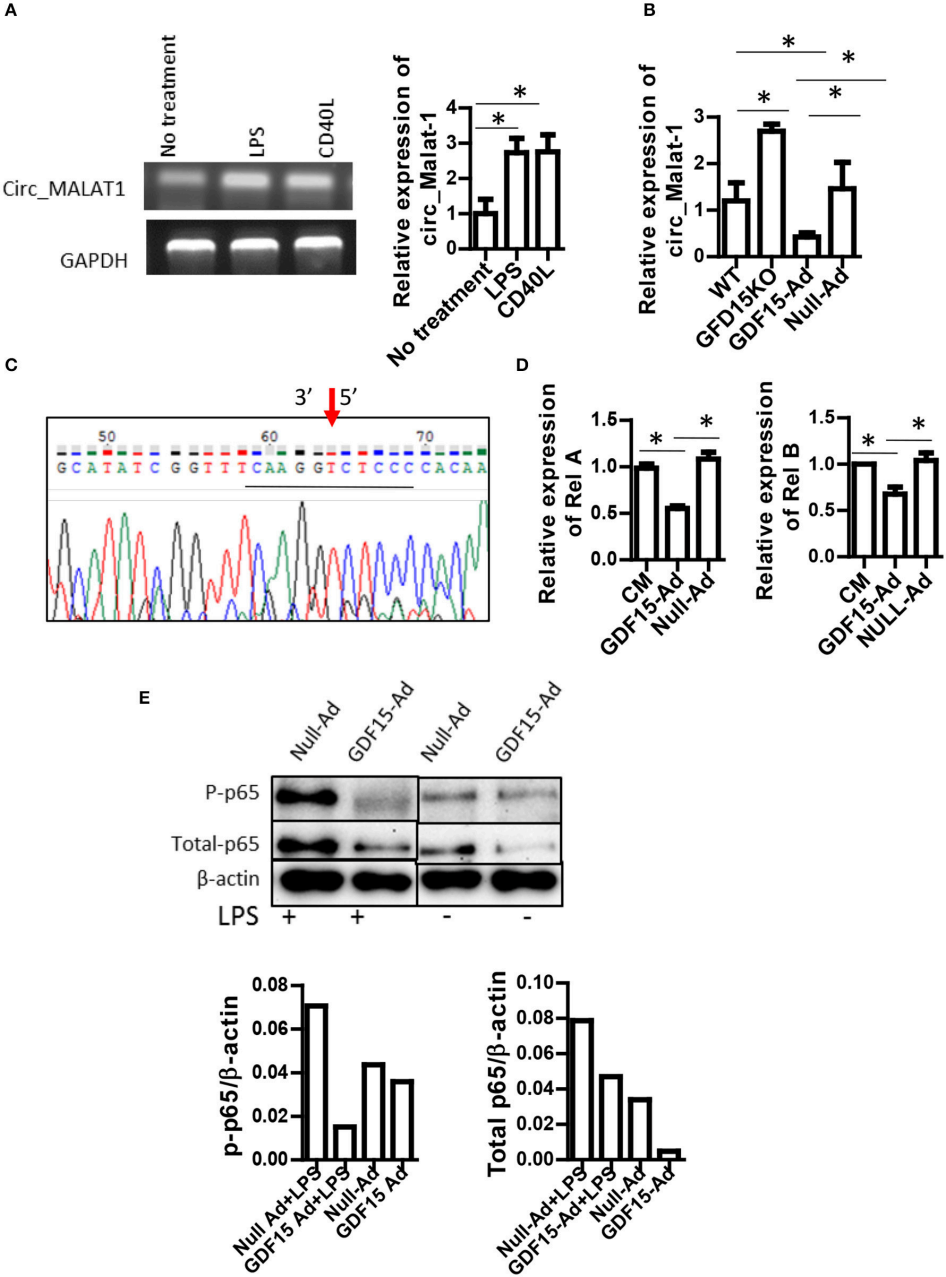
GDF15 reduced circ_Malat-1 expression and inhibited the NFκB pathway. **(A)** circ_Malat-1 expression in DCs. BM derived DCs were cultured from WT mice and treated with 50 ng/ml CD40 L or 10 ng/ML LPS for 24 h.Untreated DCs were used as control. RNA was extracted and circ_Malat-1 expression was detected by RT-PCR. Left: regular PCR, representative images for *n* = 3; Right: qPCR, *n* = 3, One way ANOVA was conducted for statistical analysis *P < 0.05. **(B)*** GDF15* negatively regulated circ_Malat-1 expression. The expression of circ_Malat 1 was detected in GDF15 KO DCs, WT DCs or GDF15-Ad or control Null-Ad treated DCs by qRT-PCR. *n* = 3, One way ANOVA was conducted for statistical analysis *P < 0.05. **(C)** Representative DNA sequence from DNA sequencing of RT-PCR products. circ_Malat-1 was amplified using divergent primers and the PCR products was then subjected to DNA sequencing using forward primer of circ_Malat-1. Arrow: pointing to the junction of circ_Malat-1. The sequencing containing the circ_Malat-1 conjunction sequence gcatatcg*gttt caaggt ctcc ccacaa* was presented. The central conjunction sequence was underlined. **(D)** The expression of Rel A and Rel B by qRT-PCR. DCs were treated with GDF5-Ad, or Null-Ad. The expression of Rel A and Rel B were measured by qRT-PCR 48 h after infection. One way ANOVA was conducted for statistical analysis *n* = 3 *P < 0.05. **(E)**. *GDF15 inhibited the NFkB signaling pathway*. Phosphorylated p65 and total p-65 protein was detected by western blotting using phosphorylated p65A Abs and p65 Abs. Representative of image of western blotting (upper) and relative quantity of protein (low) from *n* = 4 experiments. Samples treated with LPS and samples without LPS treatment were loaded separately by other samples for PAGE.

Circular RNA (circRNA) is a newly discovered non-coding RNA with 5′ and 3′ end covalent closed structure. A growing number of studies have shown that circRNA is actively involved in various physiological and pathological processes. However, it has not been reported whether circRNAs are also involved in development of DCs and in Tol-DC induction by GDF15. circ_Malat-1 was the third highly expressed circRNA in DCs and that mature DCs expressed circRNA_Malat-1 3-fold times greater than immature DCs shown in our circRNA microarray data (data not shown). Literature has reported that long non-coding RNA Malat-1, which is the parental RNA of circRNA Malat-1, regulates macrophages and DCs (27, 28). Therefore, we proposed that GDF15 may affect the expression of circ_Malat-1 expression since GDF15 inhibits DC maturation. qRT-PCR was conducted using divergent primers that can only amplify circRNA, not linear RNA, to detect circ_Malat-1. As shown in Figure 5A, DCs expressed circ_Malat-1 and both LPS and CD40 L that are stimulators for DC maturation significantly up-regulated circ-Malat-1 expression in DCs as compared with unstimulated DCs. The expression of malat-1 circular RNA (circ_Malat 1) was increased in GDF15 KO DCs as compared with WT DCs (Figure 5B). In contrast, treatment with GDF15 expressing adenovirus (GDF15-Ad) reduced circ_Malat 1 expression (Figure 5B) as compared with control adenovirus (Null-Ad). To confirm PCR specificity to circRNA, DNA sequencing was also performed on PCR products of circ_Malat-1. As shown in Figure 5C, the amplified PCR fragment contained the conjunction sequence atatcggttt caag*gt(3*′*) c(5*′*)tcc ccacaa*, confirming that the divergent primers amplified circ_Malat-1, not linear Malat-1 transcript.

Nuclear factor-kappa B (NFκB) is essential for DC maturation (29) and interruption of the pathway will impair the maturation of DCs and their function (30). There are some contradictory reports on whether GDF15 inhibits vs. activates the NFκB pathway (31, 32, 33). Nevertheless, the relationship between GDF15 and NFκB signal in DCs remains unknown. We first detected the expression of Rel A and Rel B, which are members of the NFkB family using qRT-PCR. Over-expression of GDF15 significantly reduced the expression of Rel A (Figure 5D, left) and Rel B (Figure 5D, right). We next detected phosphorylation of Rel A p65 in DCs by Western blotting. We found that the expression levels of phosphorylated p65 were decreased both in DCs treated with GDF15-Ad (Figure 5E) and in GDF15 TG DCs (Supplemental Figure 3), as compared with null-Ad treated DCs or WT DC. By contrast, phosphorylated p65 was increased in DCs from GDF15 KO mice (Supplemental Figure 3). We also found that GDF15-Ad decreased total p65 expression in DCs (Figure 5E). Taken together, the data suggested that GDF15 inhibited DC maturation through inhibition of the NFκB signaling pathway.

### GDF15 utilizes TGF-β receptors

**Figure 6:**
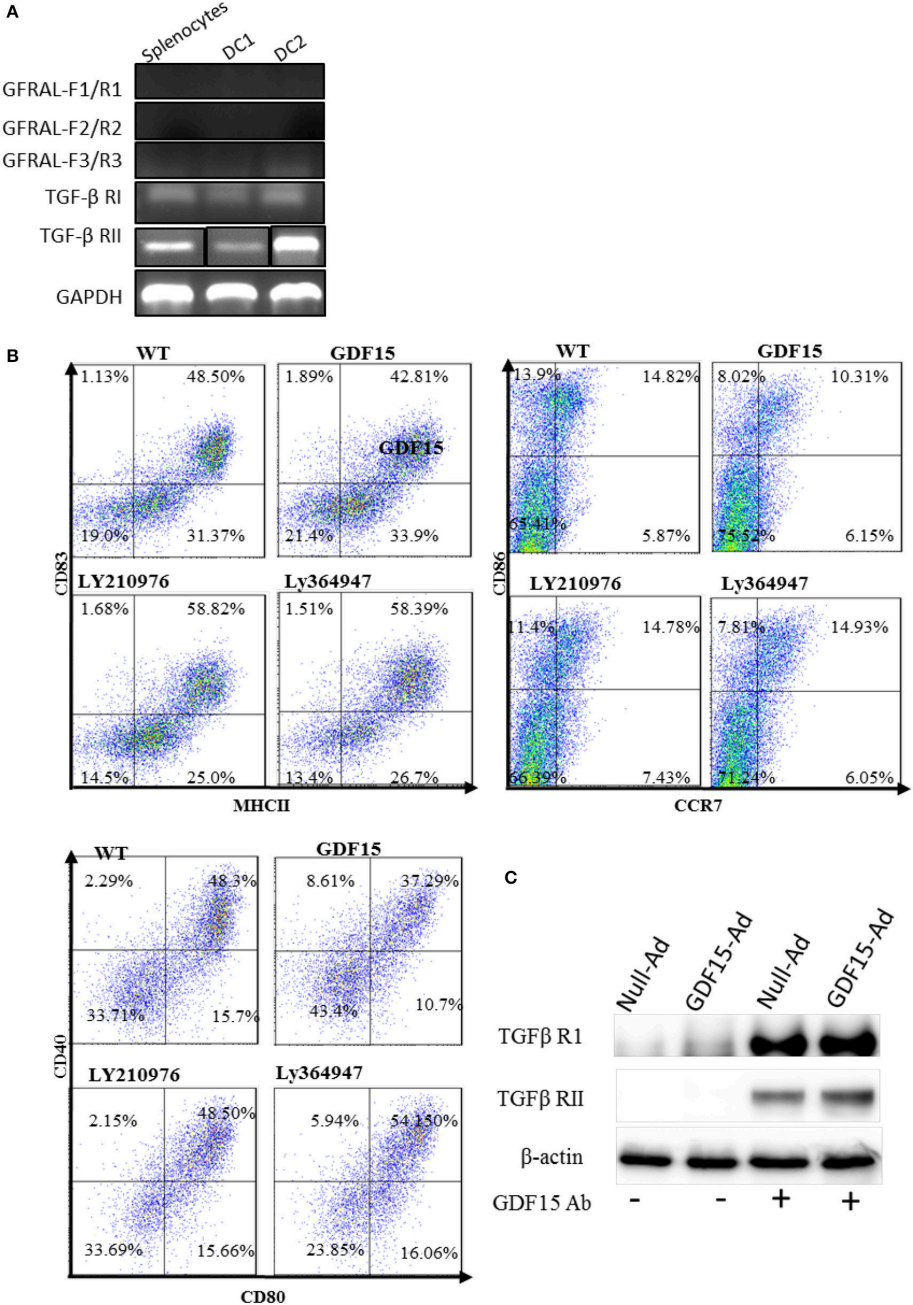
GDF15 regulated DCs by using TGFβ receptors. **(A)** Expression of GFRAL and TGF-β RI and RII. RNA was extracted from DCs and the expression of GFRAL and TGF-β RI and RII was detected by RT-PCR. PCR products were run on 1.5% agarose gel and visualized with EB. **(B)** TGF-β receptor I and II reversed the inhibitory function of GDF15 in DC maturation. DCs cultured from WT mice and treated with rhGDF15 on day 4. Ly210976 (5 mg/ml) and Ly 364947 (10 mg/ml) was added to rhgDF15 treated DCs on day 5, respectively. 24 h later, DCs maturation was determined by measuring expression of MHCII, CD40, CD80, CD83, CD86, and CCR7 flow cytometry. Data were representatives of three independent experiments. **(C)** IP assays. Total protein was extracted from the above DCs treated with GDF15-Ad or control Adenovirus and an IP assay was employed to pull down GDF15 bound protein using GDF15 Abs and control Ig Abs. IP protein was subjected to western blotting with TGFβ RI and RII Abs respectively. Representative of image of *n* = 4 experiments. PCR products for TGF-β RII from Splenocytes (SP) and two DC samples (DC1 and DC 2) were loaded separately by other genes products on an agarose gel and the image shown was modified by deletion of other genes' bands between TGF-β RII's bands.

GDF15 is a growth differentiation factor and functions through interactions with its receptor(s). It has previously been reported that GDF15 may use TGF-β receptors (TGF-β R) in non-brain tissues but this finding is contested. More recently, GDNF family receptor α-like (GFRAL), that is exclusively expressed in the brain, (17, 19) has been reported as a receptor of GDF15 used by neuron cells (34, 35). We performed RT-PCR to detect the expression of GFRAL and TGF β receptor I and II in DCs and splenocytes. Our data showed that there was no amplification of GFRAL using three pairs of primers located at three different positions in the GFRAL gene (Figure 6A), indicating that GFRAL is not expressed in both DCs and splenocytes. In contrast, DCs expressed TGF β receptor I and II (Figure 6A).

To test whether GDF15 uses TGF-β Rs, Ly364947, an inhibitor specific to TGF-β RI, and Ly2109761, a TGFβ RI and II dual inhibitor (36) were added to rhGDF15-treated DCs. The expression of MHC II co-stimulatory molecules CD40, CD80, CD83, CD86, and CCR7 was detected. As shown in Figure 6B, both Ly 364947 and Ly 2109761 resumed the expression of the aforementioned genes inhibited by rhGDF15. Data indicates that GDF15 might utilize TGF- β R I and TGF- β RII to modulate DCs.

To further confirm which receptor GDF15 utilizes, an IP assay was performed. DCs were treated with GDF15-Ad or Null-Ad on day 2 *in vitro*. On day 6, proteins were extracted from cultured DCs and GDF15 Abs were used to pull down proteins bound to GDF15, followed by Western blotting with primary Abs targeting TGF-β receptor I and II independently. We found that both TGF-β receptor I and receptor II were pulled down by GDF15 Ab (Figure 6C), although the bands for TGF receptor I were thicker than receptor II. The Data indicates that GDF15 utilizes TGF-β receptor I and receptor II for its signaling.

### GDF15-induced tol-DC prevents alloimmune rejection and prolonged allograft survival in heart transplantation

**Figure 7:**
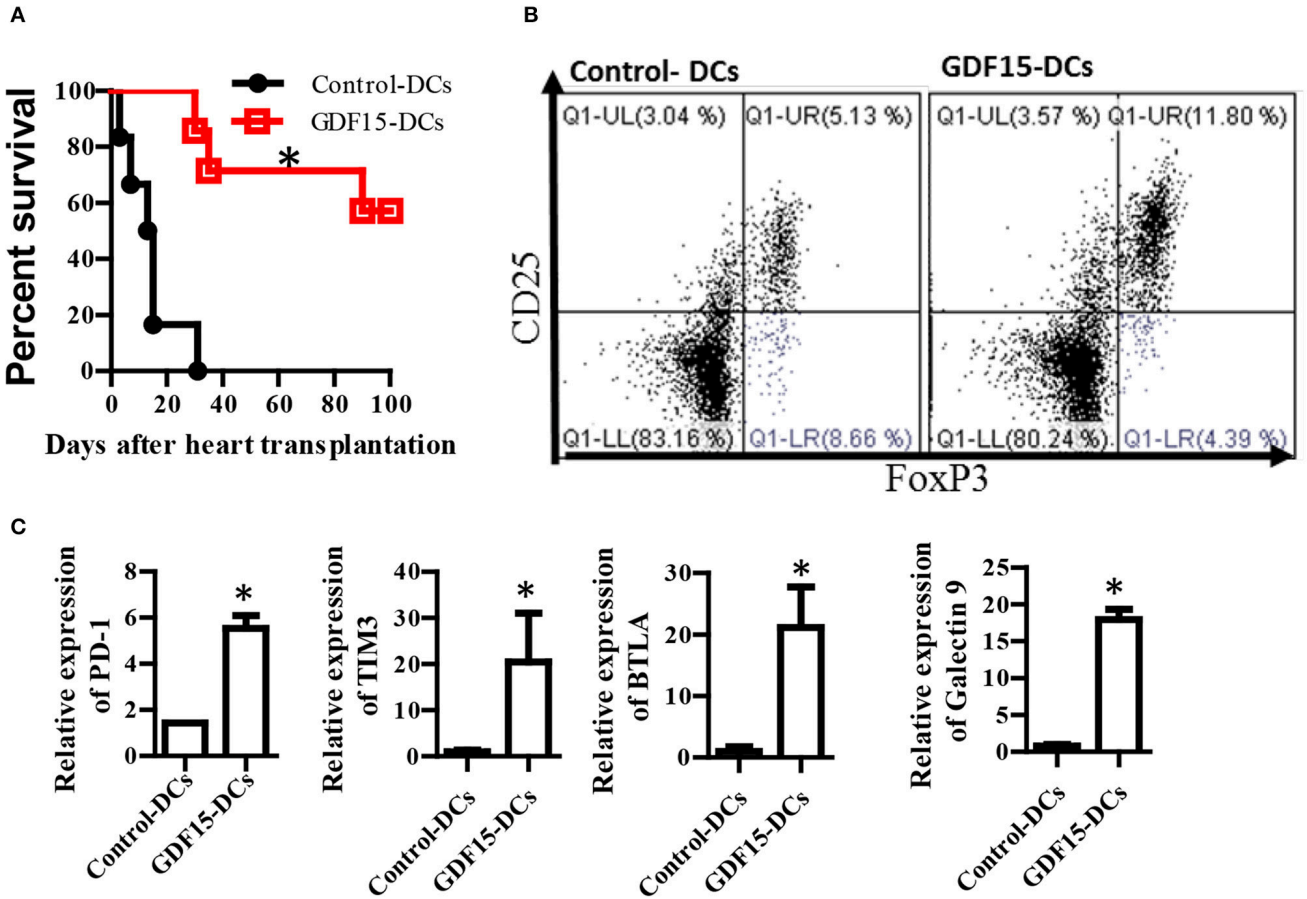
GDF15 regulated DCs prevent allograft rejection in heart transplantation and augment tolerance induction and T cell exhaustion. **(A)** Allograft survival. Donor derived (C57BL/6) DCs were cultured and treated with GDF15-Ad or Null-Ad on day 2. 1 million day 7 DCs were i.v. injected into recipient BABL/c mice 7 days prior to heart transplantation. Allogeneic heart transplantation was conducted between C57BL/6 and BABL/c mice. Recipient mice daily received 1 g/kg rapamycin for 7 days from day 0 post transplantation. The cessation of heartbeat was defined as rejection. *n* = 6. **(B)** CD4^+^CD25^+^Foxp3^+^ Tregs in splenic cells. At the end of experiments, mice splenic cells were isolated from recipient mice on day 100 to detect CD4^+^CD25^+^Foxp3^+^ Tregs by flow cytometry. Representative images (upper) and relative quantity of FoxP3 protein from *n* = 3 *P < 0.05. **(C)** Inhibitory receptor expression. RNA was extracted from the above rejected and tolerant recipient mice. Gene expression of inhibitory receptors PD-1, TIM3, BTLA and galectin 9 was detected by qRT-PCR. *n* = 3, the student *t*-test was conducted for statistical analysis *P < 0.05.

Tol-DCs have been demonstrated to be able prevent alloimmune rejection in organ transplantation (25). We investigated whether GDF15 treated DCs can prevent alloimmune rejection and immune tolerance in allogenic HT using a murine model. BM-derived DCs were cultured from donor mice and infected with GDF15-Ad on day 2. 1 million of GDF15-Ad treated DCs or Null-Ad treated DCs were intravenously injected into recipient Babl/C mice 7days prior to transplantation. Recipient mice daily received sub-dose rapamycin (1ug/kg, s.c.) from days 0 to 7 after transplantation. Heartbeat was monitored by palpation and graft rejection was defined as the cessation of heartbeat. As shown in Figure 7A, the average survival of allografts in control mice was 14 and 77.5 days for grafts in GDF15-DC treated mice. 4 allografts (two-third) in recipients treated GDF15-Ad DC still had very strong heartbeat 100 days post transplantation, indicating that GDF15-Ad DC significantly prolonged allograft survival.

At the endpoint of the experiment, CD4^+^CD25^+^FoxP3^+^ Tregs that are able to induce immune tolerance were detected. Our results show that Treatment with GDF15-DCs increased CD4^+^CD25^+^FoxP3^+^ Tregs (Figure 7B), suggesting more Treg in mice with GDF15-DCs.

T cell exhaustion has been discussed as one of mechanisms of immune tolerance (37). Our data described in Figure 4D show that GDF15 up-regulated immune inhibitory ligands and suggested that GDF15 induced exhaustive DCs that could induce T cell exhaustion. Accordingly, the expression of inhibitory receptors PD-1, Tim3, and BTAL that are highly expressed by exhausted T cells was detected. We found PD-1, Tim3, and BTAL were expressed at higher levels in splenic T cells from the immune tolerant mice treated with GDF15-Ad DCs than those from control-DCs treated rejected animals (Figure 7C), implying that GDF15-DCs induced T cell exhaustion. Induction of T cell exhaustion is another mechanism contributing to prevention of allograft immune rejection.

## Discussion

In this study, we, for the first time, reported that BM-derived DCs express GDF15 and deficiency of GDF15 accelerates DC maturation and enhances DC's immune response. We first discovered that circ-Malat-1 is differentially expressed in mature DC vs. immature DCs and that GDF15 negatively regulates circ-Malat-1 expression in DCs. We demonstrated that overexpression of GDF15 promoted exhaustive DCs to induce T cell exhaustion and enhanced Treg generation that was IDO-dependent. We also demonstrated that administration of GDF15 treated DCs prevented allograft injection and promote immune tolerance induction in HT.

GDF15 is a divergent member of the TGF-β superfamily (38). Its immunosuppressive function was first demonstrated in pregnancy, as GDF15 is highly expressed in the placenta during pregnancy and is essential to prevent rejection of the fetus (39). Zhou group reported that treatment with rhGDF15 *in vitro* prevents DC maturation and impairs the anti-tumor immune response of DCs (15). However, the authors did not address whether GDF15 is expressed by DCs, what roles endogenous GDF15 plays and how GDF215 impacts DCs at molecular level. In this study, we conducted a series of investigations on GDF15 expression in DCs. We showed that GDF15 is expressed in DCs and that its expression level is increased as DCs develop and differentiate. This increase in GDF15 expression might result from the stimulation of GM-CSF which was added to the culture medium during entire DC culture since GM-CSF has been reported to affect GDF15 expression (40). Exogenous rhGDF5 as well as GDF15-Ad treatment increased endogenous GDF15 expression in DCs and the increasing levels are dose dependent.

We further demonstrated the role of endogenous GDF15 expressed in DCs using GDF15 KO DCs or siRNA. Deficiency or knockdown of GDF15 in DCs increased the expression of MHCII, CD40,CD80 CD83, and CD86, while over expression of GDF15 either by using GDF15 TG DCs or infection with GDF15-Ad reduced the expression of these molecules, in comparison with WT untreated DCs. Our study demonstrated that exogenous and endogenous GDF15 modulated DCs toward immature DCs characterized by low expression of MHC class II and co-stimulatory molecules, and resulted DCs failed on recognition, priming and activation of T cells and presented Tol-DCs phenotype. We also found that over expression of GDF-15 led to a decrease in NFkB family members Rel A and Rel B, IL-2, IFN-γ, and IL-12p40, and an increased expression of TGF-β, and IL-10. These findings imply that GDF15 inhibited Th1-producing DCs.

We reported for the first time that over expression of GDF15 in DCs significantly up-regulated immunosuppressive genes IDO1, IDO2, and inhibitory molecules PD-L1 and galectin-9, while reducing Rel A and Rel B, and inactivating the NFκB pathway. Subsequently, these GDF15 over-expressing DCs failed in the recognition, priming and activation of T cells, thus presenting an immunosuppressive function. Moreover, over- expression of GDF15 enhanced DCs to generate CD4^+^CD25^+^Foxp3^+^Tregs which is one of the key features of Tol-DCs and IDO is necessary for Treg generation by GDF15. These findings suggest that GDF15 is able to induce Tol- DCs. Administration of these GDF15-over-expressed Tol-DCs *in vivo* successfully prevented alloimmune rejection and prolonged graft survival in HT. The current study presented a new method to induce Tol-DCs by over-expression of GDF15 and a new potential treatment for the protection of transplanted hearts from immune rejection.

The receptor GDF15 utilizes remains unclear. More recent studies have shown that GFRAL, not TGF beta receptor is the receptor for GDF15 in control of body weight loss (34, 35). Interestingly, GFRAL is exclusively expressed in brain tissue; and not in other tissues (34, 41). We detected GFRAL expression by RT-PCR and demonstrated that GFRAL was not highly expressed in DCs. This dilemma implies that there must be some other receptors GDF15 uses since GDF15 signaling was demonstrated in many other organs and non-neuron cells including DCs (15). Several early studies have suggested that GDF15 might utilize TGF-β RII and signal through canonical TGF-β receptors in heart cells (42, 43). In this study, TGF-β R inhibitors and pull-down assays were used to investigate whether GDF15 used TGF-β RII in DCs. Addition of Ly 364947, an inhibitor specific to TGF-β RI, and Ly2109761, a dual inhibitor of TGF-β RI and RII, abolished the effect of rhGDF15 on DCs. Our IP assay showed that GDF15 binds to both TGF-β R I, and RII. Taken together, our data identifies TGF-β RI and II as receptors for GDF15. TGF-β receptors could lead to the activation of multiple pathways including the NFκB, ERK, PI3K, and SMAD signaling pathways in cancer cells and heart cells (44).

The NFκB pathway is essential for DC maturation (29) and interruption of the pathway will impair the maturation of DCs and their function (30). In this study, we found that Rel A and Rel B and phosphorylation of p65 was increased in GDF15 KO DCs, but is decreased in GDF15 TG DCs and GDF15-treated DCs. The expression of AKT was increased in GDF15 over-expressing DCs and both the p38MAPK and ERK pathways seemed unaffected (data not shown). Taken together, our study demonstrates that GDF15 inhibits the NFκB pathway, resulting in the arrest of DC maturation, reduction in pro-inflammatory cytokine production, and suppression of DC immune function.

More importantly, this study, for the first time, discovered the involvement of circRNA in DC development and the link between GDF15, circ_Malat-1 and immunosuppressive function. circRNA is single stranded non-coding RNA which forms a covalently closed continuous loop (45). It is abundant, stable with a longer half- life and conserved across species. circRNAs regulate gene expression and function by acting as sponges of microRNA (miRNA) (46) and interacting with proteins (16, 17). Most circRNAs identified are generated from exons by back-splicing events and are called exonic circRNAs. circ_Malat-1 we reported here is intronic circRNA from a long non-coding RNA Malat-1, existing in the nucleus. There are no reports about circ_Malat-1. Our study shows that circ_Malat-1 was expressed in DCs at certain abundance and that circ_Malat-1 was expressed in mature DCs about 2-fold greater than in immature DCs. GDF15 significantly reduced the expression of circ_Malat-1. Taken together, circRNA is involved in DC development and circ_Malat-1 in mature DCs was significantly expressed at 2-fold higher than in immature DCs. GDF15 negatively regulates circ_Malat 1. However, the function of circ_Malat-1 needs to be further studied in the future.

In summary, we for the first time demonstrated that DCs express GDF15 and discovered a new impact of GDF15 in DC development and immune modulation such as increasing immunosuppressive molecule and immune inhibitory molecule expression, T cell exhaustion and Treg generation. We also demonstrated that administration of GDF15 regulated DCs can prevent allograft rejection and promote immune tolerance in HT. We dissected the mechanisms by which GDF 15 regulates the immune response and prevents immune rejection. On top of these findings, we first demonstrated the involvement of circRNA in DC development and GDF15 regulation of circ_Malat-1. This study also enhances the understanding of the development of mechanism-based therapies for preventing allograft rejection in transplantation. It provides a potential therapy for preventing immune rejection.

## Author contributions

YZ, GZ, YL, RC, and DZ: data collection and analysis; VM, TM, and KL: data discussion and manuscript writing; XZ: project design, data collection, analysis, and manuscript writing.

### Conflict of interest statement

The authors declare that the research was conducted in the absence of any commercial or financial relationships that could be construed as a potential conflict of interest.

## Supplementary material

Supplemental Table 1 Primer sequences for qPCR.

Supplemental Figure 1 rhGDF15 increased DC maturation. DCs (10^6^ cell/well) were cultured from bone marrow progenitors in the presence of IL-4 and granulocyte/macrophage colony stimulating factor (GM-CSF). rhGDF15 were added on day 4. DC maturation was detected by flow cytometry.

Supplemental Figure 2 Treg generation. DCs were cultured from WT, GDF15 KO, and GDF15 Tg mice. These Cs cultured for 7 days were collected and co-cultured with allogeneic naïve T cells from BABL/c mice at the ratio of 1:10 for 5 days. CD4^+^CD25^+^FoxP3^+^ cells were detected by flow cytometry. Representative images were from *n* = 3 experiments.

Supplemental Figure 3 GDF15 TG DCs expressed lower levels of Phosphorylated p65. Phosphorylated p65 and beta-actin in DCs was detected by western blotting using phosphorylated p65A Abs and beta-actin Abs. Representative of image of western blotting (upper) and relative quantity of protein (low) from *n* = 3 experiments.
