# Supplementary material for: GDF15 Regulates Malat-1 Circular RNA and Inactivates NFκB Signaling Leading to Immune Tolerogenic DCs for Preventing Alloimmune Rejection in Heart Transplantation
Source: Front Immunol. 2018 Oct 30;9:2407. doi: 10.3389/fimmu.2018.02407 (PMC6218625; doi:10.3389/fimmu.2018.02407)
Supplement: Supplemental Table 1 — Primer sequences for qPCR. [file Table_1.docx]

**Supplemental Table 1 Primer sequences for qPCR**

| Genes | Forward (5’- 3’ ) | reverse (5’- 3’ ) |
| --- | --- | --- |
| Mouse GDF15 | CGGGGCGCTGCTGTCACTTG | CCCGGGCCACCAGGTCATCATA |
| Human GDF15 | CTCCAGATTCCGAGAGTTGC | AGAGATACGCAGGTGCAGGT |
| Mouse GAPDH | GGGGTGA GGCCGGTGCTGAGTAT | CATTGGGGTAGGAACACGGAAGG |
| IL-2 | ACATTGACACTTGTGCTCCGTGTC | TTGAGGGCTTGTTGAGATGATGCT |
| IL-12 p35 | ATGATGACCCTGTGCCTTGG | CCTTTGGGGAGATGAGATGT |
| IFNγ | CCATCGGCTGACCTAGAGAAGACA | AGCCAGAAACAGCCATGAGGAAGA |
| TGFβ | AACAAACTCCACGTGGAAATCAAC | CTTGCGACCCACGTAGTAGACGAT |
| IDO 1 | GTACATCACCATGGCGTATG | CGAGGAAGAAGCCCTTGTC |
| IDO 2 | GTGGGGCTGGTCTATGAAGGTG | TGGTGGCAGCGGAGATAATGTA |
| PDL-1 | TATGGTGGTGCCGACTACAA | TGGCTCCCAGAATTACCAAG |
| Galectin 9 | GCATTGGTTCCCCTGAGA TAG | CGTTCCAGAGACCGGATCC′ |
| PD1 | GGCCGCCTTCTGTAATGGTTTGA | AGGGGCTGGGATATCTTGTTGAGG |
| TIM3 | AGTGGGAGTCTCTGCTGGGTTGA | AGGATGGCTGCTGGCTGTTGA |
| BTLA | TGCAGGAGCCAGAAGAGAAAGTCA | CAATGTGGGGGTCAGGGATGG |
| Rel B | CCGTGCCGACTTCTCTCAAG | GGGTGGCGTTTTGAACACA |
| Rel A | GCGTACACATTCTGGGGAGT | ACCGAAGCAGGAGCTATCAA |
| GFRAL- 1 | TGGGATGTTGGTTGGTGTCA | GACTGCTCACAGCCATTTGC |
| GFRAL- 2 | GGCGTTACACAGGCTGAAGA | CTGCCATGCACACAACAACA |
| GFRAL- 3 | CCCCACTTGCCTCAGTGTAA | GCAGCTCTCACTCCCAGAAC |
