# Supplementary figures and images for: GDF15 Regulates Malat-1 Circular RNA and Inactivates NFκB Signaling Leading to Immune Tolerogenic DCs for Preventing Alloimmune Rejection in Heart Transplantation
Source: Front Immunol. 2018 Oct 30;9:2407. doi: 10.3389/fimmu.2018.02407 (PMC6218625; doi:10.3389/fimmu.2018.02407)

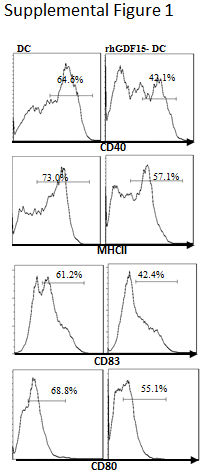

Supplement: Supplemental Figure 1 — rhGDF15 increased DC maturation. DCs (106 cell/well) were cultured from bone marrow progenitors in the presence of IL-4 and granulocyte/macrophage colony stimulating factor (GM-CSF). rhGDF15 were added on day 4. DC maturation was detected by flow cytometry. [file Image_1.tif]

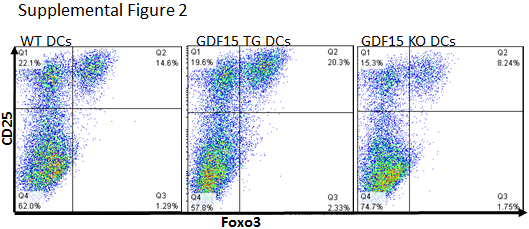

Supplement: Supplemental Figure 2 — Treg generation. DCs were cultured from WT, GDF15 KO, and GDF15 Tg mice. These Cs cultured for 7 days were collected and co-cultured with allogeneic naïve T cells from BABL/c mice at the ratio of 1:10 for 5 days. CD4+CD25+FoxP3+ cells were detected by flow cytometry. Representative images were from n = 3 experiments. [file Image_2.tif]

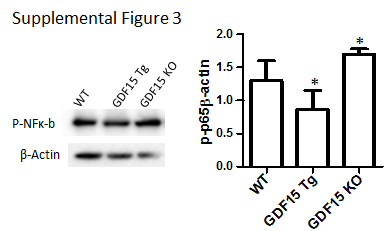

Supplement: Supplemental Figure 3 — GDF15 TG DCs expressed lower levels of Phosphorylated p65. Phosphorylated p65 and beta-actin in DCs was detected by western blotting using phosphorylated p65A Abs and beta-actin Abs. Representative of image of western blotting (upper) and relative quantity of protein (low) from n = 3 experiments. [file Image_3.tif]
